# Bleeding

**Published:** 1889-11-02

**Authors:** 


### The Practitioner's Outlook and Retrospect.

[*The Editor will be glad to receive offers of co-operation and contributions from members of the profession. There is no doubt that much valuable material full of interest and instruction is at present lost to science. This is largely so in the case of* (1) *the younger and less-known members of the hospital staff,* (2) *those connected with cottage hospitals, and* (3) *the great body of medical practitioners not attached to any institution. The co-operation of all is cordially invited to enable the Editor to make* THE HOSPITAL *of the utmost possible use and value to the profession. Specialists willing to review books on their special subjects are invited to communicate this fact at once. All letters should be addressed to* THE EDITOR, THE LODGE, PORCHESTER SQUARE, LONDON, W.]

### MEDICINE.

### BLEEDING.

"The Old Venetian Bleeding-Glass" is the title of a paper read by Dr. Knott in the medical section of the Royal Academy of Medicine, Ireland, in May last, and now published as a separate pamphlet. Dr. Knott, during a journey in Italy, possessed himself of a Venetian bleeding-glass, which had evidently belonged to some ancient noble Venetian family. The author remarks: "One easily conjures up feelings bordering on the poetic, while reflecting how much of the noble blood of Italy must have passed in and out of this modest vessel." For the Italians in extravagance of blood-letting surpassed most other nations, even in the primrose days of phlebotomy. From the records preserved to us, Dr. Knott doubts whether the Italian warrior or the surgical practitioner has had the honour of abstracting the greater quantity of the vital fluid from his fellow men. In either sex, in young and old, and in all types of disease, from the most acute to the most asthenic, large and repeated bleedings were advocated, until Van Helmont, the famous alchemist, became disgusted with the practice, because it failed to cure him of the itch! In former times when venesection formed the great keystone of treatment, princely and peasant blood fared much alike at the hands of the busy practitioner. Dr. Knott has heard old country farmers speak with deep regret of the good old days when every strong labourer prepared himself at the close of the winter for the farming operations of the spring by being "heavily blooded." Such a practice, however, was not confined to Ireland. The writer is old enough to recollect when the custom of being bled at the "rise and fall of the year" prevailed extensively in England, and also when bleeding was commonly practised for the cure of various complaints. There is a great difference between "spoliative" bleeding and moderate, or as it may be termed, "derivative" bleeding. And this appears to be Dr. Knott's opinion, for he observes for the relief of acute inflammation in the very early stage, before the elasticity of the capillary walls has been wholly lost, he would expect more tangible beneficial results from moderate bleeding than from all the other recognised remedial measures put together. "By relieving the pressure *a tergo*, it diminishes the oxidation of plasma, and as a direct consequence the cellular activity of the interstitial tissue; it thus affords an opportunity for reabsorption, and allows the capillaries to contract and regain their partially lost elasticity." Many old practitioners must recollect the relief often afforded by bleeding in painful pleurisy, for instance, or in pneumonia, or for local inflammations; and we agree with Dr. Knott that the present position of bleeding as a remedial procedure demands reconsideration by the profession.

